# Causal inference based on counterfactuals

**DOI:** 10.1186/1471-2288-5-28

**Published:** 2005-09-13

**Authors:** M Höfler

**Affiliations:** 1 Clinical Psychology and Epidemiology, Max Planck Institute of Psychiatry, Munich, Germany

## Abstract

**Background:**

The counterfactual or potential outcome model has become increasingly standard for causal inference in epidemiological and medical studies.

**Discussion:**

This paper provides an overview on the counterfactual and related approaches. A variety of conceptual as well as practical issues when estimating causal effects are reviewed. These include causal interactions, imperfect experiments, adjustment for confounding, time-varying exposures, competing risks and the probability of causation. It is argued that the counterfactual model of causal effects captures the main aspects of causality in health sciences and relates to many statistical procedures.

**Summary:**

Counterfactuals are the basis of causal inference in medicine and epidemiology. Nevertheless, the estimation of counterfactual differences pose several difficulties, primarily in observational studies. These problems, however, reflect fundamental barriers only when learning from observations, and this does not invalidate the counterfactual concept.

## Background

Almost every empirical research question is causal. Scientists conducting studies in medicine and epidemiology investigate questions like "Which factors cause a certain disease?" or "How does a certain therapy affect the duration and course of disease?" Clearly, not every association is temporarily directed, and not every temporarily directed association involves a causal component but might be due to measurement error, shared prior factors or other bias only. The only sine qua non condition for a causal effect in an individual is the precedence of the factor to its effect, and 100% evidence for causality is impossible. This insight dates back at least to the 18^th ^century Scottish philosopher David Hume [[1]; 2 chap. 1]. The question is how much evidence for a causal effect one can collect in practice and what statistical models can contribute to such evidence.

The history of causal thinking, especially in philosophy, is a history of controversies and misunderstandings. For a detailed description of these controversies, see [[1]; 2, chap. 1; [3,4]]. In this article, I argue that the counterfactual model of causal effects captures most aspects of causality in health sciences. A variety of conceptual as well as practical issues in estimating counterfactual causal effects are discussed.

The article is organized as follows: In the first two sections of the Discussion part, the counterfactual model of causal effects is defined, and some general aspects on statistical inference are discussed. The next chapters provide an overview on causal interactions and causal inference in randomised and nonrandomised studies. In the last two sections, several special topics and related approaches for assessing causal effects are reviewed.

## Discussion

### 1. The counterfactual model of causal effects

Statistics cannot contribute to causal inference unless the factor of interest *X *and the outcome *Y *are measurable quantities [3]. The temporal direction can be assessed with substantial knowledge (e.g. gender may effect diet but not vice versa) but substantial knowledge might be uncertain or even wrong. Alternatively, it can be established through the study design. Here, the causal order is ideally guaranteed by a condition in an experiment that has been manipulated before an outcome is measured [5]. If an experiment is not feasible, it is preferable to infer the temporal direction from a prospective design (e.g. a reported traumatic event at the baseline assessment as a potential risk factor for incident depression during the follow-up period) instead of collecting information on the temporal direction retrospectively in a cross-sectional study [[1]; 6 chap. 1]. Generally, in non-experimental studies, measurement error can occur not only in both *X *and *Y *but also in the assessment of their temporal direction.

To define a causal effect in an individual *i*, let us assume that we want to assess the effect of an index treatment or exposure level *t *(e.g. intake of a specific drug) as compared to another treatment or exposure level *c *(e.g. no treatment) on an outcome *Y*_*i*_. The outcome can be binary or quantitative (e.g. the amount of segregation of a hormone or a psychological score). According to Greenland and Brumback [7], we basically assume in counterfactual inference that

(a) at the fixed time point of assignment, the individual *i *could have been assigned to both treatment levels (*X*_*i *_= *t *or *X*_*i *_= *c*) and

(b) the outcome *Y*_*i *_exists under both *X*_*i *_= *t *(denoted by *Y*_*i*,*t*_) and *X*_*i *_= *c *(denoted by *Y*_*i*,*c*_).

#### Counterfactuals and potential outcomes

Obviously, the outcome can be observed only (or more precisely, at most) under one, and not under both conditions. If individual *i *is assigned to treatment level *t*, then *Y*_*i*,*c *_is unobservable; likewise, if individual *i *is assigned to treatment level *c*, then *Y*_*i*,*t *_is unobservable. The treatment that individual *i *actually does not receive is called *counterfactual *treatment. Likewise, the outcome under this treatment is referred to as *counterfactual *or *potential outcome*. The term *potential outcome *reflects the perspective before the treatment assignment and is more widespread in statistics (e.g. [8]). In contrast, the term *counterfactual outcome *denotes the perspective after the allocation; it originated in philosophy and has caught on in epidemiology (e.g. [2]). Throughout this paper, I shall use the term *counterfactual*.

A meaningful counterfactual constitutes a principally possible condition for individual *i *at the fixed time of assignment. For example, having a certain gynaecological disease instead of not having it would be an odd counterfactual condition for men. As a consequence, "influences" of intrinsic variables like sex, race, age or genotype cannot be examined with counterfactual causality in most contexts [9]. Whether "effects" of such variables should be labelled causal is controversial [7]; see [10,11] for conflicting opinions. If the discussion on causal effects, however, is restricted to those variables that might, at least in principle, be manipulated, this controversy is no longer relevant. Other factors are hardly subject to empirical research and do not serve for intervention.

In general, counterfactuals are quite natural, and, although sometimes claimed [12], there is nothing "esoteric" or "metaphysical" about them. Counterfactual reflections seem to play a vital role in creativity when human beings deal with "what would have happened if" questions [13]. In quantum physics, they have even measurable consequences [14].

#### Definition of causal effect

There is a *causal effect of treatment level *t *versus treatment level *c *in individual *i *at the time where treatment is assigned *if the outcomes differs under both conditions [e.g. [15]]:

*Y*_*i*,*t *_≠ *Y*_*i*,*c*_.

The *magnitude *of the effect can be defined in various ways: for instance, as the difference in the outcome between the two treatment levels:

*Y*_*i*,*t *_- *Y*_*i*,*c*_.

If the outcome is strictly positive, one may also use the ratio. The choice of a measure, however, affects the interpretability of a summary of individual effects as the population average effect, and the interpretability of heterogeneity of individual effect magnitudes as causal interaction (see sections 2 and 3).

To imagine a causal effect in a binary outcome suppose that an individual *i *had a particular disease. After having received a certain treatment (*X*_*i *_= *t*), the person no longer has any symptoms of the disease (*Y*_*i*,*t *_= 0). The question is whether the treatment was the cause of the remission of the disease – in comparison to another treatment level (e.g. *X*_*i *_= *c*: "no treatment"). Within the counterfactual conception, this question is equivalent to the one whether the disease would have persisted if the comparison treatment level *c *had been assigned to the same individual *i *at the same time, that is, whether *Y*_*i*,*c *_= 1. According to Maldonado and Greenland [16], this definition of a counterfactual causal effect on a binary outcome dates back to the 18^th ^century when the Scottish philosopher David Hume wrote:

"We may define a cause to be an object followed by another ... where, if the first object had not been, the second never had existed."

Counterfactual causality was the central idea that stimulated invention of randomised experiments by Ronald A. Fisher and statistical inference on them by Fisher around 1920 and, later, by Jerzey Neyman and Egon Pearson in a somewhat different way [3,17]. Much later, in 1974, Rubin [18] has firstly applied the counterfactual model to statistical inference in observational studies.

#### Choosing the reference treatment

The first difficulty in assessing counterfactual causal effects is to choose the reference condition when comparing one treatment level *t *with another treatment level *c*, that is, the substantive meaning of "treatment *c*". This does not yet constitute a real problem, because researchers should know against what alternative condition the effect of the index treatment is to be evaluated. For instance, in drug treatment trials, the effect of a drug treatment is often examined against that of a placebo treatment (*placebo-controlled trial*), because an effect resulting from the patient's impression of being treated is not the relevant kind of effect in most cases. On the other hand, if a drug has already been shown to have a positive effect, treatment with this drug may serve in comparing the efficacy of a new drug (*drug-controlled trial*). Thus, in drug-controlled trials a different effect is estimated than in placebo-controlled trials.

#### Multiple causal factors and causal mechanisms

**Figure 1:**
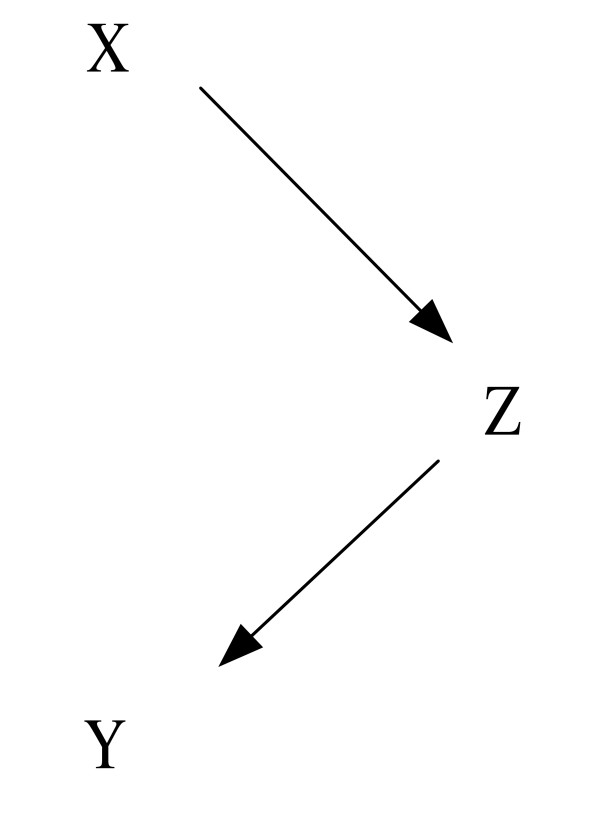
Causal graph for an indirect effect of *X *on *Y *via *Z*.

In the counterfactual model, a causal factor is a *necessary *factor without which the outcome (e.g. treatment success) would not have occurred. As the condition is not required to be sufficient for the outcome, multiple causal factors are allowed. This is in line with the fact that the etiology of most physical diseases and almost all mental disorders (e.g. [19]) is multi-causal, resulting from a complex interplay between genetical and environmental factors. Furthermore, a causal effect does not have to be a direct effect. This is desirable because an intervention like drug prescription by a doctor (if the patient complies) often causes an outcome by triggering a whole cascade of consecutive events (of biological, biochemical, mental or social origin), which, in turn, affect the outcome (directly or indirectly). In the causal graph shown in Figure 1, there is no direct effect of *X *on *Y*, but *X *causes *Y *by affecting *Z*, which, in turn, influences *Y*.

Investigating a causal effect does not require knowing its mechanism. The ability to explain an association, however, often supports the conclusion that it has a causal component (especially if the explanation is given before a researcher looks at the data). The mechanism of an effect is closely related to the terms of effect-modifying and mediating variables. An *effect-modifier *(or *moderator*) is neither affected by *X *nor by *Y *– but is associated with a "different effect of *X *on *Y*" (see section 3); a mediator is affected by *X*, and, in turn, has an effect on *Y*.

### 2. Statistical inference on counterfactual effects

As already mentioned, one can evaluate a fixed individual *i *at a fixed time only under one condition (*X*_*i *_= *c *or *X*_*i *_= *t*). Usually, no objective criteria exist to assess with a single observation whether an outcome, such as treatment success (*Y*_*i*,*t *_= 1) has been caused by the received treatment or by other factors. One exception is ballistic evidence for a bullet stemming from a particular gun and found in a killed person [20] (but here, evidence is still uncertain because the person could have died of sudden coronary failure at the moment the bullet was fired, but this possibility can be checked by autopsy). In the absence of such criteria, one can only estimate average causal effects. This requires several observations, involving different individuals or different time points or both. Many observations are also required for statistically stable conclusions.

#### Average causal effects

The aim is to estimate the *average causal effect*, that is, the average of the individual causal effects in the *target population*. The target population includes all the individuals on whom inference is to be made, whereas the population the sample is actually taken from is the *source *population [[2]; chap. 20]. Ideally, the source population equals the target population, and the individuals are randomly sampled from that population. If the sample is taken from another than the target population, selection bias will arise if the average causal effect in the source population differs from that in the target population. Moreover, the existence and magnitude of different biases (e.g. confounding [21], see below) depend on the choice of target population, and information on biases stemming from populations other than the target population might not apply.

To be interpreted as an estimate of the population average effect, the difference between the arithmetic mean in *X *= *t *versus *X *= *c *(summary over all individuals in the respective treatment group) has to equal the arithmetic mean of the differences at the level of the individuals. Linear differences can always be interpreted in this way [22], whereas for multiplicative measures like the mean ratio and the risk ratio the geometric mean has to be used instead. The population average interpretation of the summary odds ratio, however, becomes increasingly false with an increasing number of individuals at high risk for the outcome (under one or both conditions) [22].

The following discussion is restricted to the more frequent case of a sample consisting of different individuals rather than of different time points (or both).

#### Stable-unit-treatment assumption

Before treatment assignment, there are two random variables for each individual *i *in the population: the outcome under treatment *c *(*Y*_*i*,*c*_) and the outcome under treatment *t *(*Y*_*i*,*t*_). Although the theory can be extended accordingly [23], I shall now assume for simplicity that the outcomes of individual *i *are independent of the outcomes of other individuals and their received treatment levels. This is referred to as the *stable-unit-treatment*-assumption [23]. Note that this might be a quite restrictive assumption: it does not hold for contagious diseases as outcome. Influenca is such a disease in which the immunisation of certain individuals may affect the others (called "herd effect", e.g. [24]). After treatment assignment and the observation of the outcome, a sample of *n *individuals contains (at most) one realisation of the outcome for each individual *i *where the outcome corresponds either to treatment level *t *or *c*. Therefore, from the statistical point of view, the estimation of causal effects can be regarded as a particular problem of missing values (e.g. [17]).

#### Exchangeability

Suppose the average causal effect is defined as the difference in means in the target population between both conditions *X *= *t *and *X *= *c*. Then the simplest way to estimate it is with the difference between the two sample means (denoted by  and , resp.). If individuals with *X *= *c *and *X *= *t *are "exchangeable", average causal effects can be directly estimated as  without bias due to assignment (bias might exist anyway due to other causes such as measurement or selection). *Exchangeable *means that two conditions have to be fulfilled [21,25]:

a) The distribution of the unobserved outcome *Y*_*t *_under actual treatment *c *is the same as that of the observed outcome *Y*_*t *_under actual treatment *t*; that is, under counterfactual treatment with *t*, the individuals actually treated with *c *would behave like those actually treated with *t*; individuals having received treatment *t *are *substitutes *for individuals having received treatment *c *with respect to *Y*_*t*_.

b) The distribution of the unobserved outcome *Y*_*c *_under actual treatment *t *is the same as that of the observed outcome *Y*_*c *_under actual treatment *c*; that is, under counterfactual treatment with *c*, the individuals actually treated with *t *would behave like those actually treated with *c*; individuals having received treatment *c *are *substitutes *for those who have received treatment *t *with respect to *Y*_*c*_.

Note that, if individuals actually having received treatment *c *and *t*, respectively, correspond to different populations and inference is to be made solely on one of these two populations, then only either assumption a) or b) is required. For instance, if inference is to be made only on the population having received treatment *c*, condition a) is sufficient.

In the section on causal inference, I will provide an outline on how exchangeability relates to different study designs and what statistical methods can contribute to approach unbiased estimation of causal effects if the optimal design (a perfect randomised experiment) is not feasible.

### 3. Heterogeneity in causal effects

An important issue is the assessment of differences in causal effects between individuals. Clearly, a necessary condition for a factor *Z *to be a modifier of the effect of *X *on Y is that *Z *precedes the outcome *Y*. If such a potential effect-modifier *Z *is associated with *X*, the parameter that describes the modification of the effect of *X *on *Y *is not identified without making further assumptions. Effect-modifiers are typically assessed with interaction terms in regression models.

#### Choice of the effect measure

Whether and, if yes, to what extent the degree of an effect differs according to the values of *Z *depends, however, on the choice of the model and the associated index of effect magnitude. As mentioned above, some effect measures (e.g. the odds ratio) usually serve only to quantify the magnitude of a causal effect supposed to be constant between the individuals.

Moreover, the risk difference is the only measure for which effect heterogeneity is logically linked with causal co-action in terms of counterfactual effects. To explain this, it is necessary to define the causal synergy of two binary factors, *X*_*i *_and *Z*_*i *_(coded as 0 or 1), on a binary outcome *Y*_*i *_in an individual *i *(at fixed time).

Clearly, if *X*_*i *_and *Z*_*i *_do not act together in causing the event *Y*_*i *_= 1, then

(a) if *Y*_*i *_= 1 is caused by *X*_*i *_only,

   *Y*_*i *_= 1 if (*X*_*i *_= 1 and *Z*_*i *_= 0) or

      (*X*_*i *_= 1 and *Z*_*i *_= 1)

and *Y*_*i *_= 0 in all other cases. Thus, *Y*_*i *_= 1 occurs in all cases where *X*_*i *_= 1 and in no other cases.

(b) if *Y*_*i *_= 1 is caused by *Z*_*i *_only,

   *Y*_*i *_= 1 if (*X*_*i *_= 0 and *Z*_*i *_= 1) or

      (*X*_*i *_= 1 and *Z*_*i *_= 1)

and *Y*_*i *_= 0 in all other cases. Thus, *Y*_*i *_= 1 occurs in all cases where *Z*_*i *_= 1 and in no other cases.

Therefore, causal synergy means that 1) *Y*_*i *_= 1 if either one or both factors are present and 2) *Y*_*i *_= 0 if neither factor is present. Now, one is often interested in *superadditive *risk differences, where the joint effect of *X *= 1 and *Z *= 1 is higher than the sum of the effects of (*X *= 1 and *Z *= 0) and (*X *= 0 and *Z *= 1) as compared to the risk for *Y *= 1 under (*X *= 0 and *Z *= 0), that is,

P(*Y *= 1 | *X *= 1, *Z *= 1) > P(*Y *= 1 | *X *= 1, *Z *= 0) + P(*Y *= 1 | *X *= 0, *Z *= 1) - P(*Y *= 1 | *X *= 0, *Z *= 0).

If superadditivity is present, one can show that there must be causal synergy between *X *and *Z *on *Y*, at least for some individuals [[2], chap. 18; [26,27]]. This relation does not apply in the opposite direction: If there is causal synergy among some individuals there may be no superadditivity. Thus, one can demonstrate rather a causal interaction than its non-existence. Note that other logical relations do not exist and the risk difference is the only measure for which such a logical link exists [[2]; chap. 18; [26,27]]. Also, other measures like correlations, standardised mean differences or the fraction of explained variability do not serve to quantify the degree of causal effects because they mix up the herefore solely relevant mean difference with parameters of exposure and outcome variability [28].

Another crucial point for the choice of effect index is whether the interaction terms in regression models corresponds with so-called *mechanism-based *(e.g. biological) interactions [29]. For instance, if the dose of intake of a particular drug is known to influence the release of a certain hormone linearly, then the interaction term of another factor with drug intake in a linear model corresponds to the presence of a biological interaction.

#### Deterministic versus probabilistic causality

A fundamental question relating to heterogeneity in causal effects is the distinction between deterministic and probabilistic causality [[2], chap. 1; [30], chap. 1]. The functional-deterministic understanding of causality is based on the Laplacian conception of natural phenomena, which are assumed to follow universally valid natural laws. Here, in the absence of measurement error and other biases, the observable heterogeneity in *Y *– given *X *and the other observed covariates – would be attributed solely to unobserved factors. If we knew the causal mechanism completely (how complicated it may be) and the values of all the causal factors, the outcome *Y *would be exactly determined. Note that I have implicitly used this assumption in the previous discussions.

Within the probabilistic understanding of causality, individual variation exists within the outcome *Y*, which can not be explained by unconsidered factors. This variation might be called *real randomness *and can be found in quantum physics [14]. It is possible to incorporate real randomness into counterfactual models because one can specify a probability distribution for a potential outcome of a fixed individual at a fixed time [[7] and references therein]. In real situations, however, the distinction between deterministic and probabilistic causality does not play a major role in systems that are complex enough for substantial residual heterogeneity in the modelled effect to be expected. Here, the effect is practically probabilistic. Such a situation is rather the rule than the exception in medical and behavioural sciences.

On the other hand, after incorporating major effect-modifiers into a model, the effect of *X *on *Y *should be sufficiently homogeneous to allow for uniform interventions in the subpopulations defined by the values of the effect-modifiers. As a consequence of the existence of effect-modifiers, a variation in their distribution across different populations implies that one would expect to estimate different effects if the modifiers were not considered in a model. Thus, differences in estimates of effects do not imply that different causal mechanisms act; instead, they might be solely due to different distributions of hidden effect-modifiers [[2], chap. 18; [16]]. Interactions with intrinsic variables; that is, individuals' immutable properties like sex, race and birth date are often regarded as an indication of a narrow scope of a model [31]. On the other hand and as mentioned above, nonmanipulable properties are hardly subject to counterfactual arguments.

### 4. Causal inference in randomised and non-randomised studies

#### Randomised experiments

As already mentioned, if the individuals are exchangeable between the treatments and there are no other biases, causal effects can be directly estimated, most simply with the difference in the mean of *Y *between *X *= *c *and *X *= *t*. A stronger assumption than exchangeability is related to the *propensity score*. The propensity score is the probability of individual *i *being assigned to treatment *t *– at the time when group assignment to *X *= *c *or *X *= *t *takes place, denoted with *PS*_*i *_= P (*X*_*i *_= *t*). The assumption that the propensity score is equal among the individuals with *X *= *c *or *X *= *t *is stronger than the assumption of exchangeability because the determinants of the propensity score do not necessarily affect the outcome *Y*.

In a simple randomised experiment, *PS*_*i *_is equal for all individuals. For example, in an experiment with balanced groups, the individuals are assigned to each treatment with a probability of 50%: *PS*_*i *_= 1/2 for all *i*. More sophisticated designs incorporate a covariate or, more generally, a vector of covariates *Z*into the group assignment (*block designs*). Provided that such covariates are also factors of the outcome, considering them often yields increased statistical precision in the estimate of the causal effect. In randomised experiments, the propensity score is a known function g of the realisations *z*of *Z*; that is, *PS*_i _= g(*z*_i_) and the joint distribution of *Y*_*c *_and *Y*_*t *_is conditionally independent of *X *given *Z*, a property called *strong ignorability *[8]. Now, one can show that *X *and *Z*are conditionally independent given the propensity score; that is, the propensity score *PS *summarises all information contained in *Z*about the group assignment [8]. As a consequence, the mean effect of *X *on *Y *can be approximatively estimated without bias due to assignment if the entities are matched pairwise according to the propensity score, if they are weighted proportionally to the inverse propensity score, or if the propensity score is adjusted for in a suitable regression model [8]. From a Bayesian perspective, the estimates of the propensity score are posterior probabilities to predict the allocation to exposure (*X *= *t*) under *Z *= *z *[32]. The problem with the propensity score is that it is sufficient to control for but not minimally sufficient (it may include unnecessary information due to covariates related to *Y *but not to *X*).

#### Imperfect experiments

In the discussion above I have implicitly assumed that treatment and control protocols were followed exactly; in that sense, the experiments were supposed to be perfect. In many studies, however, the actual treatment and control conditions do not equal the intended protocols, at least, not for some individuals or measurement points (*imperfect *or *broken experiments*). For instance, in the pharmacotherapy of depression with antidepressants, one often faces the problem that many individuals in the antidepressant treatment group (*X *= *t*) stop drug intake as, in the beginning, they experience only adverse effects [33]. According to Imbens and Rubin [34], imperfect experiments constitute the bridge between experiments with ideal compliance and observational studies.

#### Instrumental variables

If one ignores the fact that the treatment conditions were not exactly followed, one estimates the effect of the intended, not of the actual treatment. This is referred to as *intent-to-treat analysis*. Alternatively, one can estimate the effect of the treatment *among those who complied*. This can be done with approaches based on *instrumental variables*. Roughly speaking, an instrumental variable *I *is a variable that is associated with the actual treatment or exposure *X *but that related to the outcome *Y *only through its association with *X*. Maybe the most important example for an instrumental variable is the intended treatment. The basic idea of such approaches is that one can – under certain conditions that vary with the specific problem – compute the *X *- *Y *association or bounds of it from the *I *- *X *and the *I *- *Y *association [35,36]. These methods are useful when the observed *X *- *Y *association is more confounded than the *I *- *X *and the *I *- *Y *associations. Another situation where instrumental variable methods apply is when not *X *but only a surrogate *I *of it can be directly observed. The association between *I *and *X *then has to be known or estimable, and differences between *I *and *X *have to be independent of other variables [35,36].

#### Observational studies

Not every interesting factor can be translated into equivalent lab settings or can be manipulated. Factors like social support or peer relationships are difficult to observe outside their natural environment. Other conditions should not be assigned to human beings for ethical reasons (e.g. smoking). In such cases, there is no way but to conduct an observational study. In observational studies, the group assignment is neither manipulated nor randomised. The group status *X *is a random variable subject to measurement error, and the individuals assign themselves to *X *= *c *or *X *= *t*, for example, by deciding to smoke or not to smoke.

The propensity score then typically depends on a variety of variables (denoted as vector *Z*). Often, not all of such factors are observable or even known. Researchers conducting epidemiological and nonrandomised clinical studies should aim at collecting data on the major determinants of *X *to allow for an adequate control of confounding. Note that variables associated with *X *but not with *Y *can often be ignored. However, they can serve to reduce the variance in normally distributed outcomes, but adjusting for them sometimes yields unnecessarily high variances in outcomes with other distributions [8].

In many practical situations, one should assume substantial residual bias due to unobserved determinants of the exposure *X*, which, in turn, affect *Y*. Such kind of bias is referred to as *confounding*. A *confounder *is a variable that is associated with both *X *and *Y *and that precedes *X*; and adjusting for it reduces the overall bias in the estimation of the causal effect of *X *on *Y *[[2], chap. 15]. In practice, however, it is not determinable whether a certain variable is a confounder because this depends on all (other) confounders and biases together. If *Z*_*l *_is a candidate for a confounder, the difference between the means under *X *= *t *and *X *= *c *adjusted for *Z*_*l *_might be biased more strongly than the unadjusted mean difference. This can happen, for instance, if other, more important factors of group assignment are distributed more unequally across *X *= *c *und *X *= *t *after stratification than they were before stratification on *Z*_*l *_[[7], and the references therein].

Pearl [[30], chap. 3; [37]] has discovered formal criteria within the framework of graphical models (the "backdoor" and "frontdoor" criterion, resp.) that indicate which set of covariates is sufficient to be controlled for. Applying these criteria, however, requires assumptions on the causal system that causes *X *and *Y*. Some of the variables that cause *X *and *Y*, in turn, are often unobserved or even unknown.

#### Methods to adjust for unobserved confounding and other biases

There are various approaches to address unobserved confounding, bias due to measurement error, selection, and other biases. The first method is sensitivity analysis, which examines what impact one or several supposed scenarios of bias would have had on the results at hand. The results depend on the presumed values of bias parameters like misclassification probabilities, the distribution of a confounder, and the magnitude of it's effects on *X *and *Y*. For a general model for sensitivity analyses, see [38]. Rosenbaum [39] has proposed a general framework to assess how sensitive a particular study design is against assignment bias. The problem with sensitivity analysis is that only the range of expected results under different specified values for the unknown bias parameters is revealed [40].

This drawback is solved with Monte Carlo sensitivity analysis. Here, distributions are assigned to the unknown bias parameters, which reflect a researcher's knowledge or assumptions about their true values. Bias-corrected point and interval estimates can then be calculated. The results from these methods have approximatively a Bayesian interpretation if additional uncertainty is added (as would be the case if one drew random numbers from the posterior distribution of the unknown effect), the estimator of the causal effect is approximatively efficient, and the data provide no information on the bias parameters [[40] and references therein].

(Monte Carlo) sensitivity analyses and Bayesian methods outperform conventional analyses, which often yield overconfident and biased results because they are based on wrong point priors at zero (e.g. misclassification probabilities) at the parameters determining bias [40]. This is true as long as the assumptions made are not fundamentally wrong (e.g. bias downward instead of upward, [41]). In conventional analyses, the farther the left boundary is from the null, the more room there is for bias and extra-variation. Moreover, a statistically significant difference does not imply that the association found is strong enough to be of a clinical or policy concern; the absence of a statistically significant association often does not even rule out a strong relation (e.g. [[2], chap. 12; [42]]). Hence, it is essential to quantify the degree of association also in perfect randomised experiments and to report an interval estimate.

### 5. Some more special issues

#### Time-varying exposures

In many applications, the exposure level *X *is not a constant condition but a sequence of treatment levels (*generalised *or *g-treatment*) that varies within individuals over time. For instance, Robins et al. [43] have investigated the effect of prophylaxis therapy for pneumocystis carinii pneumonia (PCP, an opportunistic infection in AIDS patients) on survival times among AIDS patients in an uncontrolled study. In medical studies, the exposure level often varies over time, for example, because physical complications require a change in treatment or because individuals deposit drug intake because of adverse effects.

The problem with time-varying systems is that they are subject to feedback mechanisms: The causes at fixed time *q *might not only be affected by causes of the outcome occurring before time *q *(confounding), but they may also impact later time-dependent causes [44,45]. For instance, the outcome at time *q*-1 may be a mediator for the outcome at time *q*, but a confounder of the exposure at time *q*. As in the above example statistical inference for causal effects of time-dependent exposures is often based on survival time as outcome and, therefore, on survival models. The associated methods easily become complicated because one often has to take several issues into account. These include measured and unmeasured confounder adjustment, feedback mechanisms and censoring (not all individuals are observed throughout the whole investigation time). Then they still share all the limitations of conventional methods in observational studies (bias due to measurement, selection etc., [45]).

Details of statistical models are rather technical and thus beyond the scope of this paper. Briefly, Robins [44] has derived a general recursive *g-computation *algorithm, from which he has derived non-parametrical tests. These tests, however, turned out to be impractical for inference more sophisticated than simple null hypothesis testing (e.g. [45]). Later, more flexible semiparametric models (called g-estimation) of survival outcomes were developed (e.g. [43]). These models make assumptions merely on the form of the difference between the treatment levels rather than on the shape of the outcome distribution within the same treatment (and covariate) level. An alternative approach is provided by so-called "marginal structural models" and "inverse-probability of treatment-weighted estimators". In the case of censoring, these methods are less complex than g-estimation at the cost of requiring stronger assumptions here [45]. However, they often allow for improved confounder adjustment [46]. Gill and Robins [47] have developed extensions of g-estimation for continuous time.

#### Competing risks

Suppose that one is interested in the health burden attributable to a variable that is actually an outcome and not a treatment action in the earlier sense. Let me borrow an example from Greenland [48]: Suppose one is interested in how the number of years lived after the age of 50 (*T*) is affected by whether smokers died of cancer (*Y *= 1) or not (*Y *= 0). Assume that a certain individual *i *was a male lifetime heavy smoker and died from lung cancer at the age of 54 (*T*_*i *_= 4 | *Y*_*i *_= 1). Now the estimation of *T*_*i *_under *Y*_*i *_= 0 is unclear because it depends on how *Y*_*i *_= 0 was caused, how death from lung cancer was prevented. If death by lung cancer had been prevented through convincing the individual not to smoke at all in his entire lifetime, then the risk of other causes of death (e.g. coronary heart disease, diabetes, or other kinds of cancer) would be lower as well. In this case, *T*_*i *_under *Y*_*i *_= 0 might be considerably higher than 4 years. On the other hand, if *Y *= 0 was caused by chemotherapy, the risks of other diseases, named *competing risks *[[48] and references therein] would not have been reduced. Hence, the outcome *T*_*i *_under *Y*_*i *_= 0 might not have been much higher here than under *Y*_*i *_= 1. The expected increase in years lived would thus be much smaller if lung cancer was prevented by chemotherapy than it would be if lung cancer was prevented by lifetime absence of smoking.

To conclude, there is no single intervention in this case that would be independent of an individual's history prior to exposure. The evaluation of the effect of removing *Y*_*i *_= 1 depends on the *mode *of removal in a multivariate framework. Therefore, effects of policies should be evaluated in terms of actions that cause outcome removal rather than in terms of outcome removal per se [48].

#### The probability of causation

A common problem is how to determine the probability that an event in an individual has been caused by a certain exposure, that is, the *probability of causation *(*PC*). Courts define causation as an exposure without which the outcome event would a) not have happened at all or b) have happened later. Such a cause is named *contributory cause *[49]. The empirical basis for an estimate of the probability of causation in an individual is a sample of exposed individuals. This sample should be similar to the individual under investigation with respect to the history of exposure and (other) risk factors of disease. Then, one can estimate the rate fraction (*RF *– often called "attributable fraction"), the excess incidence rate due to exposure – relative to the incidence rate if exposed, given by



where *IR*_*X *= 0 _and *IR*_*X *= 1 _denote the incidence rates in the target population under exposure and under non-exposure, respectively [[2]; chap. 3]. The *etiological fraction *(*EF*) is defined as the fraction of exposed individuals with the disease for which the exposure was a contributory cause of the disease [[2]; chap. 3]. Now, the probability of causation in the individual equals the etiological fraction (*PC *= *EF*) if the individual was randomly drawn from the target population [49]. A common fallacy, however, is to confuse the rate fraction *RF *with the probability of causation *PC *(in the sense of a contributory cause). To illustrate this mistake algebraically, one can express the etiological fraction as



where:

- *C*_1 _is the number of individuals in the population in which exposure has caused an *accelerated onset *of disease (i.e., under non-exposure, the disease would have occurred anyway but later);

- *C*_2 _denotes the number of individuals in whom exposure has caused *all-or-none *disease (i.e., without exposure, these persons would not have contracted the disease at all); and

- *C*_*T *_is the total number of persons exposed to the disease (including also those individuals who have not been affected by the exposure, [49]).

Now, one can show that, if the probability of the exposure having an effect in the exposed is low, the rate fraction *RF *approximately equals *A*_2_/*A*_*T *_[49] – a quantity known as the excess rate [[2], chap. 4]. Thus in this case, the equation *PC *= *RF *approximatively holds only if *A*_1 _is small as compared to *A*_2_. This means that the effect is required to have an all-or-none effect in the vast majority of exposed and diseased individuals. Otherwise, the probability of causation is underestimated proportionally to the ratio *A*_1_/*A*_2_. A fundamental problem with the estimation of *PC *is the estimation of *A*_1 _– the number of exposed and diseased persons who would have developed the disease later under non-exposure. This estimation would require some biological model (which seems to be rarely available) for the progress of the disease [49]. Robins and Greenland [50] have provided upper and lower limits for the probability of causation that are consistent with the data. Pearl [51] showed under which conditions the probabilities that a factor was a necessary or a sufficient cause, respectively, can be estimated from the data.

### 6. Related approaches to causal inference

#### The sufficient-component-cause model

Rothman [52] has proposed a model of causal effects that is similar to but finer than the counterfactual model — the *sufficient-component-cause model*. Entities in this model are not individuals but mechanisms of causation. A mechanism is defined as a combination of factors that are jointly sufficient to induce a binary outcome event, *Y *= 1. Each of possibly many of such mechanisms has to be minimally sufficient: The omission of one factor would change *Y *from 1 to 0; that is, the outcome event would no longer be present. For instance, following an example by Rothman [52], it is not sufficient to drink contaminated water to get cholera; other factors are required as well. If, in this example, drinking contaminated water is part of each mechanism that leads to cholera, this constitutes a *necessary *factor for cholera. For a fixed individual at a fixed time, often several mechanisms are in line with the same counterfactual effect [[2], chap. 18; [7]]. Therefore, the sufficient-component-cause model is important rather for conceptional than for inferential considerations. Rothman's [52] intention was to build a bridge between metaphysical reflections and epidemiological studies.

#### Structural equation models

Especially in the fields of psychology, social sciences and economics, structural equation models (SEMs) with latent variables are frequently used for causal modelling. These models consist of (a) parameters for the relations among the latent variables, (b) parameters for the relations among latent and observed variables and (c) distributional parameters for the error terms within the equations. Pearl [30] has shown that certain nonparametric SEMs are logically equivalent to counterfactual models and has demonstrated how they can be regarded as a "language" for interventions in a system. Furthermore, these models are useful to structure and reduce variance, for example, to reduce measurement error if several items on a questionnaire are assumed to represent a common dimension.

There are, however, several practical problems with the use of SEMs. First, in an under-determined system of equations, several assumptions are necessary to identify the parameters (i.e. to make the estimates unique). In psychological applications, the assumptions tend to be justified only partially [53] and models with alternative assumptions are often not considered [54]. The results, on the other hand, may be very sensitive against these assumptions [55], and currently, there is no way to model uncertainty in these assumptions. Besides, the coefficients from these models are sometimes not interpretable as measures of conditional dependencies (i.e. regression coefficients), for instance, if there are loops in a model [56]. Finally, the meaning of the latent variables remains sometimes obscure, and — in economic applications — results from certain structural equation models have been found to fail to recur in experiments [57].

It is therefore recommended that one should be extremely careful in the application of SEMs. For more sophisticated discussions of the relations among structural equation models, graphical models, the corresponding causal diagrams and counterfactual causality; see [7,30,31,58] and the papers cited therein.

#### The controversy on counterfactual causality raised by Dawid's article [12]

Dawid [12] has argued that counterfactuals were something metaphysical because causal inference based on counterfactuals would depend on unobservable assumptions. In his own formulation of the counterfactual model, Dawid assumed that a causal effect in an individual was composed of the average effect of treatment *t *versus *c*, an individual effect and an interaction term treatment*individual. Different assumptions about the unidentified individual parameters would yield different conclusions about the variance of the counterfactual effect. Such assumptions involved the joint distribution of *Y*_*c *_and *Y*_*t *_for fixed individuals.

Together with Dawid's paper in the Journal of the American Statistical Association, not less than seven commentaries as well as Dawid's rejoinder [59] were published. Cox [60] reproached Dawid for posing too general a question and for going much too far with his conclusions: The proof of a causal effect would not require knowing its mechanism. Shafer [61], on the other hand, regretted that David had been too mild in condemning counterfactuals. Casella and Schwarz [62] mentioned that every scientific investigation had to aggregate over different individuals. Pearl [63] and Cox [60] argued that, in contrast to Dawid's claims, several aspects of counterfactual causality were at least indirectly testable. Wasserman [64] pointed out that, as in every other kind of statistical models, the identifiability of parameters would be essential in causal models but that counterfactuals provided a quite useful conception. Robins and Greenland [65] brought up the point that Dawid had largely neglected observational studies and imperfect experiments. Probabilistic causal inference (of which Dawid is an advocate) in observational studies would inevitably require counterfactuals. Otherwise, causal effects may not be identified without again making unidentified assumptions. Rubin [66] considered the modelling of the joint distribution of *Y*_*c *_and *Y*_*t *_as not always necessary.

Dawid [12] rejects the counterfactual concept seemingly because, on it's own, it is not powerful enough to solve the fundamental problems of causal inference (e.g. in a fixed individual at a fixed time one can observe the outcome only under one condition). Depending on the question and the design, there are indeed often unidentified parameters. I argue that the fact that the concept does not solve all problems does not mean that it is wrong; in that sense, denying the usefulness of counterfactuals is as if a doctor never prescribed a drug that may not remedy all his patients, but several of them. Counterfactual causal thinking is based on imagining the consequences of changing the value of a single factor in a comprehensive causal system. What would the world look like after changing the value of one variable (in one or several individuals) is what some philosophers of science call the *possible worlds *concept of causality [15] (see also [[30], chap. 7] for a formal definition). Our imagination of possible worlds, however, always depends on substantive knowledge required to formulate a causal system that might have produced the data that one has observed. This, though, does not mean that we should not ask for properties of possible worlds because the decisions we aim to conduct (e.g. which interventions to make) depend on these unknown properties.

## Summary

1. The counterfactual concept is the basis of causal thinking in epidemiology and related fields. It provides the framework for many statistical procedures intended to estimate causal effects and demonstrates the limitations of observational data [10].

2. Counterfactual causality has also stimulated the invention of new statistical methods such as g-estimation.

3. The intuitive conception makes the counterfactual approach also quite useful for teaching purposes [65]. This can be exemplified by illustrating the difference among study designs. For instance, the benefit of longitudinal over cross-sectional studies is easily demonstrated when the aim is to study how several variables act together over time when causing an outcome.

4. Counterfactual considerations should replace vague conceptions of "real" versus "spurious" association, which occasionally can still be read. In this context, the Yule-Simpson paradox is often mentioned. This paradox indicates that an association can have a different sign (positive or negative association, resp.) in each of two different subpopulations than it has in the entire population. However, if the temporal direction of the variables is added to this paradox and there is no bias and random error, the paradox is resolved: It is then determinable which association is *real *and which is *spurious *in a causal sense.

5. Causal effects have been treated like a stepchild for a long time, maybe because many researchers shared the opinion that causality would lie outside what could be scientifically assessed or mathematically formalised. Pearl [30,37] was the first to formulate the difference between changes in variables induced by external intervention in a system and changes due to variation in other variables in the system.

## Competing interests

The author(s) declare that they have no competing interests.

## Pre-publication history

The pre-publication history for this paper can be accessed here:


